# Temporomandibular Joint Osteoarthritis Diagnosis Employing Artificial Intelligence: Systematic Review and Meta-Analysis

**DOI:** 10.3390/jcm12030942

**Published:** 2023-01-25

**Authors:** Oana Almășan, Daniel-Corneliu Leucuța, Mihaela Hedeșiu, Sorana Mureșanu, Ștefan Lucian Popa

**Affiliations:** 1 Department of Prosthetic Dentistry and Dental Materials, Iuliu Hațieganu University of Medicine and Pharmacy, 400006 Cluj-Napoca, Romania; 2 Department of Medical Informatics and Biostatistics, Iuliu Hațieganu University of Medicine and Pharmacy, 400349 Cluj-Napoca, Romania; 3 Department of Oral and Maxillofacial Surgery and Implantology, Iuliu Hațieganu University of Medicine and Pharmacy, 400029 Cluj-Napoca, Romania; 4 2nd Medical Department, Iuliu Hațieganu University of Medicine and Pharmacy, 400006 Cluj-Napoca, Romania

**Keywords:** temporomandibular joint, osteoarthritis, artificial intelligence, systematic review

## Abstract

The aim was to systematically synthesize the current research and influence of artificial intelligence (AI) models on temporomandibular joint (TMJ) osteoarthritis (OA) diagnosis using cone-beam computed tomography (CBCT) or panoramic radiography. Seven databases (PubMed, Embase, Scopus, Web of Science, LILACS, ProQuest, and SpringerLink) were searched for TMJ OA and AI articles. We used QUADAS-2 to assess the risk of bias, while with MI-CLAIM we checked the minimum information about clinical artificial intelligence modeling. Two hundred and three records were identified, out of which seven were included, amounting to 10,077 TMJ images. Three studies focused on the diagnosis of TMJ OA using panoramic radiography with various transfer learning models (ResNet model) on which the meta-analysis was performed. The pooled sensitivity was 0.76 (95% CI 0.35–0.95) and the specificity was 0.79 (95% CI 0.75–0.83). The other studies investigated the 3D shape of the condyle and disease classification observed on CBCT images, as well as the numerous radiomics features that can be combined with clinical and proteomic data to investigate the most effective models and promising features for the diagnosis of TMJ OA. The accuracy of the methods was nearly equivalent; it was higher when the indeterminate diagnosis was excluded or when fine-tuning was used.

## 1. Introduction

According to Cohen S., the term “artificial intelligence” (AI) is still a little confusing [1]. Artificial intelligence was initially described in 1956 by implementing specific learning algorithms in computers to effectively manage human issues [2]. Artificial intelligence applications are available in almost any medical and nonmedical area, increasing their presence in healthcare as a consequence of their broad use of big data and progressively changing the way practitioners approach disease [3].

Machine learning (ML) belongs to a class of computer algorithms that build models for characterizing and forecasting using previously known data [1].

In dentistry, AI is used in multiple areas; from determining the influence of dental aesthetics on facial attractiveness [4], intraoral scanning [5], forecasting post-operative skeletal changes in orthognathic surgical planning [6], maxillary sinus segmentation [7], early detection of oral cancer [8], alveolar bone segmentation from cone-beam computed tomography (CBCT) [9], obtaining fully automated cephalometric measurements from a web-based artificial intelligence-driven platform [10], assessing root position during orthodontic treatment [11], introducing algorithms in dentomaxillofacial radiology [12], diagnosing an anteriorly displaced temporomandibular joint (TMJ) disk on magnetic resonance imaging (MRI) [13], and diagnosing TMJ disorders [14] or TMJ osteoarthritis [15,16].

Osteoarthritis (OA) is a major and severe disorder that has generally been accepted as a whole-organ disease or a combination of diseases [17]. It is described as the chronic destruction of the soft and hard tissues around joints, frequently associated with cartilage damage, bone remodeling, synovitis, and joint discomfort [18]. OA of the TMJ was found to affect 25% of the adult population (20 to 50 years) when clinical signs were sought along with MRI investigations [19], whereas in older patients, its prevalence increases drastically to 70% [20]. Osteoarthritis of the TMJ is one of the most frequent degenerative joint disorders [21,22] and is characterized by condyle flattening, resorption, osteophyte formation [23], and degenerative alterations of the articular eminence, such as erosion, sclerosis, or resorption [24,25,26].

The insufficiency of signs before severe joint destruction occurs renders the early diagnosis of TMJ OA difficult [22]. Therefore, diagnosing TMJ osteoarthritis efficiently and precisely is key to effective treatment planning. Furthermore, the significant prevalence of TMJ OA underlines the necessity for a comprehensive imagistic evaluation of this condition, especially using modern AI techniques.

To the best of our knowledge, we could not identify any systematic review assessing the use of AI in TMJ OA.

Thus, the aim of our paper was to systematically synthesize the current research and the influence of AI models on TMJ OA diagnosis using CBCT or panoramic radiography.

## 2. Materials and Methods

The systematic review was reported in accordance with the recommendations of the “Preferred Reporting Items for Systematic Reviews and Meta-Analyses Protocols (PRISMA) Statement” [27]. The systematic review has been registered in the open science framework and can be found at the following address: https://osf.io/qnzd5/ (accessed on 31 December 2022).

### 2.1. Eligibility Criteria

All publications on osteoarthritis of the temporomandibular joint that considered artificial intelligence as a diagnosis method were included. Exclusion criteria were considered case reports, systematic reviews, narrative or scoping reviews, abstracts, comments, communications, editorials, and letters to the editor.

### 2.2. Information Sources

In May 2022 we performed a structured electronic search in the following databases: PubMed, Embase, Scopus, Web of Science, LILACS, ProQuest, and SpringerLink. Where applicable, MeSH and Emtree terminology were employed. The last electronic search was performed on all databases on 28 May 2022.

### 2.3. Search Strategy

The following terms were used in the search strategy: “osteoarthritis”, “degenerative joint disease”, “temporomandibular joint”, “temporomandibular joint disorders”, “artificial intelligence”, “machine intelligence”, “machine learning”, “deep learning”, “supervised”, “unsupervised”, “support vector machines”, “random forest”, “classifier”, “classification algorithm”, “cross validation”, “data mining”, “feature detection”, “feature extraction”, feature learning”, “feature selection”, “k nearest neighbor”, “pattern recognition”, “KNN”, “K-means”, “principal component analysis” “XGBoost”, “LightGBM”, “neural network”, “tensorflow”, “PyTorch”, “Keras”, “ResNet”. Search terms included synonyms, acronyms, and singular as well as plural form words. In Table 1, the full strategies adjusted for each database are shown.

### 2.4. Selection Process and Data Collection Process

The search had no time constraint, nor were there any search limits or filters. The online Endnote version was used to remove double entries [28], followed by manual removal. A Microsoft Excel file (Microsoft Office 365, MS, Redmond, WA, USA) [29] was used to organize the publications after all of the papers had been retrieved and to carry out an impartial, blind screening of the included studies. The selection was carried out independently by two researchers (O.A. and D.C.L.). When unsure whether to include a particular study, the researchers conferred with two more researchers to find their standpoint (S.M. and S.L.P.). The same authors independently evaluated the chosen articles for inclusion after accessing the full texts, with disagreements being settled through debate. Two reviewers (S.M. and S.L.P.) collected data from the articles in a predefined Excel form file [29]. Inadvertences were compared with the full-text article by a third and fourth author (M.H. and D.C.L.). The following data were acquired: (1) author and year of publication; (2) study population; (3) OA classification; (4) training, validation, and testing; (5) region of interest (ROI) extraction; (6) transfer learning models; (7) learning; (8) software; and (9) results. These data are presented in Supplementary Table S1. Version 6.0.6 of the Zotero software (Roy Rosenzweig Center for History and New Media, Fairfax, VA, USA) was used to manage all references [30].

### 2.5. Study Risk of Bias Assessment

Two reviewers (O.A. and D.C.L.) independently judged the methodological quality of each of the chosen articles; any discrepancies in their evaluations were then compared in order to reach a consensus. The QUADAS 2 risk of bias assessment (Table 2) [31] and the minimum information about clinical artificial intelligence modeling (MI-CLAIM) checklist (Table 3) [32] were used to study the risk of bias.

### 2.6. Effect Measures

The sensitivity and specificities of the AI classification of TMJ OA by human experts were computed for each study.

### 2.7. Synthesis Methods

OpenMeta {Analyst} software was used to perform the meta-analyses. We extracted the true positives, false positives, false negatives, and true negatives from each study. The sensitivity and specificity were computed using the random-effects model with the restricted maximum likelihood estimator and presented in forest plots. The heterogeneity of the meta-analysis results was assessed with I^2^ and the χ2-based Q-test and qualified using the Cochrane Handbook recommendations [33]. For all results, the point estimator, 95% confidence intervals, and *p*-values were presented. A 0.05 level of significance was used for all statistical tests.

### 2.8. Reporting Bias Assessment

The publication bias assessment is inconsequential since there were few identified studies.

## 3. Results

### 3.1. Study Selection

**Figure 1:**
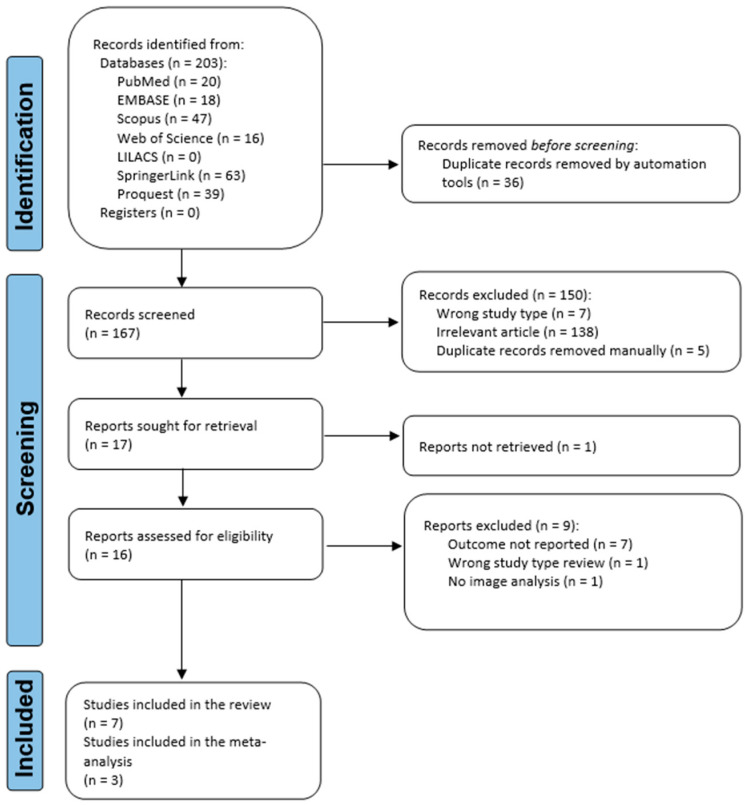
Flowchart of the identification, screening, and inclusion of articles in the systematic review.

A PRISMA flow diagram was used to portray the recruiting and selection process (Figure 1). A total of 203 records were identified from seven databases: PubMed, Embase, Scopus, Web of Science, LILACS, ProQuest, and SpringerLink. After removing duplicate records, 167 records were screened. Out of these, 150 were excluded from the screening process. Seventeen publications were sought for retrieval, but one was not retrieved, although it was requested by email from the corresponding author. Out of the articles assessed for eligibility, nine studies were excluded. Seven articles were included in the qualitative and quantitative synthesis, amounting to 10,077 TMJ images, of which, the meta-analysis included three studies, amounting to 5520 TMJ images.

### 3.2. Study Characteristics

The study characteristics are presented in Supplementary Table S1. We grouped the studies according to the imaging diagnosis techniques in CBCT [16,34,35,36] and panoramic radiography [15,37,38].

Bianchi J. [34] tested the diagnostic performance of four machine learning models: Logistic Regression, Random Forest, LightGBM, and XGBoost, trained on 52 features (clinical features (age, years of pain, vertical range unassisted and without pain, and others), 20 radiomics features (e.g., energy, entropy, bone volume, trabecular thickness, and others), and 14 serum and saliva biomarkers) and several interactions, finding that the XGBoost + LightGBM model achieved the highest accuracy of 0.823, AUC 0.870, and F1-score of 0.823 to diagnose the TMJ OA.

De Dumast P. [35] built a web-based system for storing, integrating, and computing biomedical data. They constructed 3D surface models from the CBCT and then applied a shape variation analyzer, a deep neural network classifier for osteoarthritis of the temporomandibular joint, to achieve a 91% agreement between the clinician and the SVA classifier.

Lee K.S. [36] constructed a diagnostic tool that uses artificial intelligence, a single-shot object detection model, to automatically identify normal, indeterminate TMJ OA, and TMJ OA in CBCT images. Their results, including indeterminate TMJ OA diagnosis vs. excluding them, were an average precision = 0.80 vs. 0.89, set average recall = 0.77 vs. 0.90, and F1 score = 0.78 vs. 0.89.

Zhang W. [16] used the same subjects as Bianchi J. [34] but used Learning using Privileged Information (LUPI) on 77 features (6 clinical, 46 imaging, and 25 protein) and interactions, finding that the LUPI method outperformed non-LUPI methods.

Choi E. [37] created an AI model and assessed the performance of the model using OPGs’ TMJ OA diagnostics against an oromaxillofacial radiology (OMFR) specialist. Using a Karas’ ResNet pre-trained model, an AI model was created and trained to divide panoramic radiography images into three groups: normal, uncertain OA, and OA. Results for the testing set including indeterminate TMJ OA diagnosis vs. excluding them were an accuracy = 0.51 vs. 0.78, weighted average precision = 0.55 vs. 0.78, weighted average recall = 0.51 vs. 0.78, and F1 score = 0.53 vs. 0.78.

Jung W. [15] created a diagnostic aid by categorizing panoramic images of TMJ into normal and osteoarthritis instances using pre-trained transfer learning models. ResNet-152 vs. EfficientNet-B7 accuracy, sensitivity, specificity, and area under the curve (AUC) values were 0.87, 0.94, 0.79, and 0.94, vs. 0.88, 0.86, 0.91, and 0.95.

Kim D. [38] used ResNet and Inception V3 pre-trained models and Visual Geometry Group-16 convolutional neural networks (CNNs) to suggest an algorithm that can extract the condylar area and assess its irregularity. The results concerning accuracy (ac.), sensitivity (Se), specificity (Sp), and AUC, without vs. with fine-tuning were: VGG16 ac. = 0.78 vs. 0.84, Se = 0.49 vs. 0.54, Sp = 0.86 vs. 0.94, AUC = 0.76 vs. 0.82; ResNet ac. = 0.77 vs. 0.81, Se = 0.41 vs. 0.47, Sp = 0.77 vs. 0.91, AUC = 0.57 vs. 0.79; Inception V3 ac. = 0.79 vs. 0.82, Se = 0.39 vs. 0.41, Sp = 0.82 vs. 0.94, and AUC = 0.51 vs. 0.83.

Concerning ROI identification, three studies used manual selection [16,34,36]. Jung W. [15] started with an automated tool, followed by manual selection of the ROI. De Dumast P. [35] segmented the CBCTs to create 3D surface models, and all condylar models were concurrently cropped to obtain the ROI. Choi E. [37] used a faster RCNN using the Inception V3 model to generate region proposals for the ROI. For each region, feature vectors were derived using Inception ResNet V2r, and an SVM predicted the class, followed by a bounding box regression for accurate object location. Kim D. [38] used an R-CNN to detect the TMJ and joint fossa and condyle, followed by a CNN to detect abnormalities based on the shape of the TMJ.

### 3.3. Results of Syntheses

**Figure 2:**
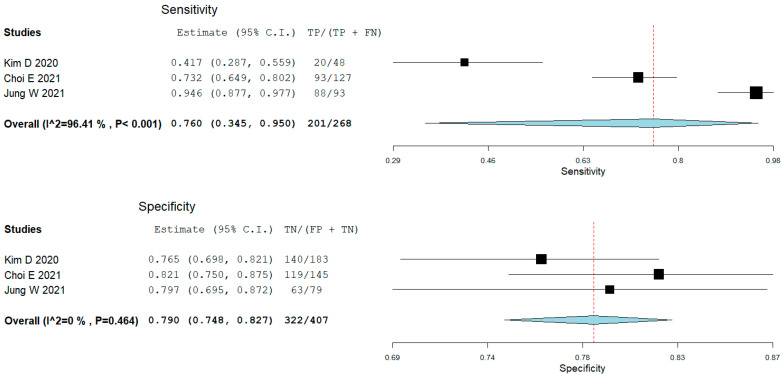
Forest plot for sensitivity and specificity of ResNet in classifying temporomandibular joint osteoarthritis (Choi [36], Jung, [16], Kim [37]).

From the studies that assessed panoramic radiography with AI, three studies presented the results of the ResNet classifications of TMJ OA; all studies excluded indeterminate TMJ OA diagnosis. We performed a meta-analysis of the test results without fine-tuning the models (Figure 2). The pooled sensitivity was 0.76 (95% CI 0.35–0.95), *p* = 0.208. The heterogeneity between the studies’ results was considerable (I2 = 96.4%, *p* < 0.001). The pooled specificity was 0.79 (95% CI 0.75–0.83), *p* = 0.208. Though the heterogeneity between the studies’ results might not be important (I2 was 0%, *p* = 0.464).

### 3.4. Risk of Bias Assessment in Studies

**Figure 3:**
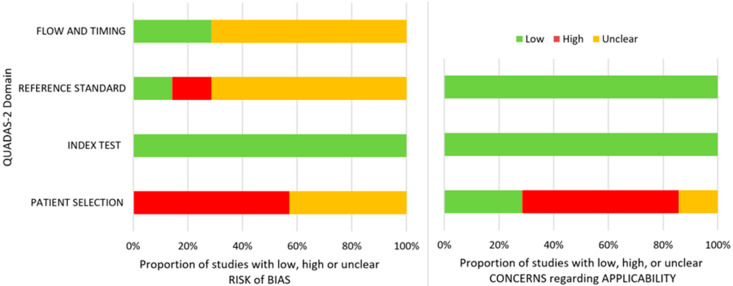
QUADAS 2 risk of bias and overall applicability assessment.

The detailed QUADAS 2 risk of bias and applicability assessment is presented in Table 2 and Figure 3. We used two questions for this review that were assessed with the QUADAS 2 tool: for studies [15,37,38,39] of patients with TMD-related symptoms (without comorbidities that may influence the TMJ diagnosis) who are assessed with imagistic methods (panoramic radiography or CBCT), how accurate may an AI predict TMJ OA?; while for studies [16,34,35] of patients with TMD-related symptoms (without comorbidities that may influence the TMJ diagnosis) who are assessed with imagistic methods (any method) and other features (clinical and biomolecular), how accurate may an AI predict TMJ OA?

**Table 2:** QUADAS 2 risk of bias assessment.

Criteria	Choi * [36]	Jung * [16]	Kim * [37]	Lee * [34]	Bianchi # [32]	De Dumast # [33]	Zhang # [35]
Patient selection							
Signaling questions							
Was a consecutive or random sample of patients enrolled?	unclear ^a^	unclear	unclear	unclear	unclear	unclear	unclear
Was a case-control design avoided?	yes	yes	yes	yes	no	unclear	no
Did the study avoid inappropriate exclusions?	yes	yes	yes	yes	yes	unclear	yes
Risk of bias assessment	unclear	high ^d^	high ^d^	unclear	high	unclear	high
Applicability	low	low	high ^e^	high ^f^	high ^g^	unclear	high ^h^
Index test							
Signaling questions							
Were the index test results interpreted without knowledge of the results of the reference standard?	yes	yes	yes	yes	yes	yes	yes
If a threshold was used, was it pre-specified?	NA	NA	NA	NA	NA	NA	NA
Risk of bias assessment	low	low	low	low	low	low	low
Applicability	low	low	low	low	low	low	low
Reference standard							
Signaling questions							
Is the reference standard likely to correctly classify the target condition?	unclear ^b^	unclear ^b^	no ^i^	yes	unclear ^b^	unclear ^b^	unclear ^b^
Were the reference standard results interpreted without knowledge of the results of the index test?	yes	yes	yes	yes	yes	yes	yes
Risk of bias assessment	unclear	unclear	high	low	unclear	unclear	unclear
Applicability	low	low	low	low	low	low	low
Flow and timing							
Signaling questions							
Was there an appropriate interval between index test(s) and reference standard?	yes	yes	yes	yes	yes	yes	yes
Did all patients receive a reference standard?	yes	yes	yes	yes	yes	yes	yes
Did patients receive the same reference standard?	unclear ^c^	unclear ^c^	unclear	yes	unclear	yes	unclear
Were all patients included in the analysis?	yes	yes	yes	yes	yes	yes	yes
Risk of bias assessment	unclear	unclear	unclear	low	unclear	low	unclear

*, Risk of bias assessment question: For patients with TMD-related symptoms (without comorbidities that may influence the TMJ diagnosis) who are assessed with imagistic methods (panoramic radiography or CBCT), how accurate may an AI predict TMJ-OA? ^#^ For patients with TMD-related symptoms (without comorbidities that may influence the TMJ diagnosis) who are assessed with imagistic methods (any method) and other features (clinical and biomolecular), how accurate may an AI predict TMJ-OA? ^a^, Symptoms + OPG + CBCT; ^b^, no information about experience, reliability; ^c^, multiple specialists; ^d^, excluded indeterminate diagnosis; ^e^, dental treated patients; ^f^, all diagnosed with TMD and TMJOA on CBCT; ^g^, excluded symptoms >= 10 years or important destruction; ^h^, used resubstitution validation; ^i^, CBCT not used for diagnosis but orthopantomography; NA, not applicable; TMJ, temporomandibular joint; TMD, temporomandibular disorder; AI, artificial intelligence; CBCT, cone beam computed tomography; OA, osteoarthrosis; and QUADAS 2, quality assessment of diagnostic accuracy of studies.

Regarding the patient selection domain, four studies had a high risk of bias due to the exclusion of indeterminate diagnoses or due to the use of a case-control design; the other three studies had an unclear risk of bias. The index test domain was assessed to have a low risk of bias for all the studies. In connection with the reference standard, one of the studies had a low risk of bias, one had a high risk of bias, and five had an unclear risk of bias because the authors did not provide information about the experience of the image evaluators, and the reliability of the assessments. With reference to the flow and timing, five of the studies had an unclear risk of bias since multiple specialists may have performed the assessments of the TMJ diagnosis, and two had a low risk of bias.

In respect of applicability in the patient selection domain, four studies had a high risk of bias since some studies included treated patients, had all patients with TMD, or excluded patients with important destruction or long TMJ symptomatology. Next, one study had an unclear risk, and the other one had a low risk. Concerning the index test and reference standard, the risk was low.

The minimum information about the clinical artificial intelligence modeling (MI-CLAIM) checklist was used to assess the selected articles, and its results are presented in Table 3.

**Table 3:** The minimum information about the clinical artificial intelligence modeling (MI-CLAIM) checklist.

Study Design (Part 1)	Choi [36]	Jung [16]	Kim [37]	Lee [34]	Bianchi [32]	De Dumast [33]	Zhang [35]
The clinical problem in which the model will be employed is clearly detailed in the paper.	yes	yes	yes	yes	yes	yes	yes
The research question is clearly stated.	yes	yes	yes	yes	yes	yes	yes
The characteristics of the cohorts (training and test sets) are detailed in the text.	yes	yes	no	yes	yes	no	yes
The cohorts (training and test sets) are shown to be representative of real-world clinical settings.	yes	no	no	no	no	no	no
The state-of-the-art solution used as a baseline for comparison has been identified and detailed.	yes	unclear	unclear	yes	unclear	unclear	unclear
Data and optimization (Parts 2, 3)							
The origin of the data is described, and the original format is detailed in the paper.	yes	yes	no	yes	yes	no	yes
Transformations of the data before it is applied to the proposed model are described.	no	no	no	no	yes	yes	yes
The independence between the training and test sets has been proven in the paper.	yes	yes	yes	yes	yes	yes	yes
Details on the models that were evaluated, and the code developed to select the best model are provided.	yes *	yes *	yes *	yes *	yes *	yes *	yes *
Is the input data type structured or unstructured?	uns	uns	uns	uns	both	both	both
Model performance (Part 4)							
The primary metric selected to evaluate algorithm performance (e.g., AUC, F-score, etc.), including the justification for selection, has been clearly stated.	yes ^a^	yes ^a^	yes ^a^	yes ^a^	yes ^a^	no	yes
The primary metric selected to evaluate the clinical utility of the model (e.g., PPV, NNT, etc.), including the justification for selection, has been clearly stated.	yes ^a^	yes ^a^	yes ^a^	yes ^a^	yes ^a^	no	yes
The performance comparison between the baseline and the proposed model is presented with the appropriate statistical significance.	yes	yes ^b^	yes ^b^	yes ^b^	yes ^b^	yes ^b^	yes
Model examination (Part 5)							
Examination technique 1a	no	no	no	no	no	no	no
Examination technique 2a	no	no	no	no	no	no	no
A discussion of the relevance of the examination results with respect to model/algorithm performance is presented.	yes	yes	yes	yes	yes	no	yes
A discussion of the feasibility and significance of model interpretability at the case level if examination methods are uninterpretable is presented.	NA	NA	NA	NA	NA	NA	NA
A discussion of the reliability and robustness of the model as the underlying data distribution shifts is included.	no	no	no	no	no	no	no
Reproducibility (Part 6): Choose the appropriate tier of transparency							
Tier 1: Complete sharing of the code.	no	no	no	no	yes	yes	no
Tier 2: Allow a third party to evaluate the code for accuracy/fairness; share the results of this evaluation.	no	no	no	no	no	no	no
Tier 3: Release of a virtual machine (binary) for running the code on new data without sharing its details.	no	no	no	no	no	no	no
Tier 4: No sharing.	yes	yes	yes	yes	no	no	yes

NA, not applicable; *, no code for automatic selection of the models—they were chosen by the authors; uns, unstructured; ^a^, no justification; and ^b^, no statistical test.

Study design: The clinical problem and research question were clearly stated in all the papers. The characteristics of the cohorts were not clearly detailed in two articles. The cohorts were not clearly representative of real-world clinical settings in six articles. State-of-the-art being used as a baseline for comparison was unclear with respect to the experience of the image evaluators.

Data and optimization: The origin of the data was not clearly described in two articles. Three articles performed transformations of the data before applying the model. All the papers described the independence between the training and the test sets and they gave the details on the models that were employed. Four studies used unstructured data (images), while three used both structured (clinical and biological data) and unstructured data (images). One study did not clearly present the primary metric to assess the algorithm performance and clinical utility; nevertheless, they presented the confusion matrix. All the papers provided a performance comparison between the baseline and the proposed model.

Model examination: No study showed sensitivity analyses nor a discussion of the reliability and robustness of the model as the underlying data distribution shifts are included. Only one study did not discuss the relevance of the examination results with respect to model performance.

Reproducibility: Only two studies shared their code.

## 4. Discussion

Our exhaustive research of the literature identified several articles concerning TMJ OA classification with AI that were described and assessed for methodological quality. A meta-analysis was then applied to the studies that used ResNet for panoramic radiography assessment.

Two studies checked the diagnostic performance of several machine learning models on a large number of features (clinical, radiomics on CBCT, and proteomics from serum and saliva) [16,34] in an exploratory approach, with XGBoost + LightGBM being the most accurate, as well as LUPI methods, outperforming by a small margin the non-LUPI methods. One study used a single-shot detector deep learning framework designed for object detection on CBCT [36]. Another study reconstructed the 3D shape of condyles and used a shape variation analyzer to classify TMJ OA in five different morphological degeneration groups [35]. Three other studies assessed the pre-trained transfer learning models (ResNet, EfficientNet, VGG, and Inception V3) on panoramic radiographs [15,37,38], with the fine-tuned VGG model being the most accurate in the head-to-head comparison (on 2584 images [38]), but yielded small differences between them. Since all three studies assessed the ResNet model, we performed a meta-analysis to synthesize their results (on 5520 images). The pooled sensitivity was 0.76 (95% CI 0.35–0.95) with marked heterogeneity. The outlier study here was that of Kim D. [38], with a sensitivity of 0.42. This value was for a model without fine-tuning and could explain the difference. It is possible the other studies did not specify if they did or did not fine-tune their results. The pooled specificity was 0.79 (95% CI 0.75–0.83) with low heterogeneity. The overall accuracies or sensitivities and specificities are not very impressive, being clinically moderate. We must keep in mind that panoramic radiography is not the primary intention diagnosis test when it comes to TMJ imaging. CBCT, on the other hand, is more accurate in diagnosing the bone pathology of TMJ; however, in the selected studies, we could not identify similar studies using this imaging technique to perform a meta-analysis. It is difficult to compare the accuracies of AI classifying on panoramic radiography and CBCT since they were not trained on the same images, but the expectancy would be that AI trained on CBCT would outperform those trained on panoramic radiography.

Several studies excluded indeterminate TMJ OA diagnoses. This exclusion artificially increases the accuracies, as can be seen in the results of several studies. The use of AI in real-life scenarios would have lower diagnostic accuracies.

As expected, fine-tuned models outperformed the models without hyperparameter tweaks.

The selection of the ROI influences the accuracy of the training since a poorly chosen ROI cannot offer good discriminant information for the AI. Almost half of the studies used manual ROI selection that can offer high-quality training data, but this suffers from the pipelining of AI in real-life scenarios. One study combined an automated tool with manual selection. Three studies applied CNNs to generate, and another CNN to predict, the ROI, with the most sophisticated approach being the one used by Choi E. [37].

### 4.1. Limitations

The number of images used in several studies was low, nevertheless, the models had important accuracies (possibly due to the use of pre-trained models and data augmentation methods). The exclusion of indeterminate diagnoses or illegible and blurry images artificially increased the model accuracies in several studies. The exclusion of subjects with a history of orthognathic surgery, craniofacial trauma, and systemic diseases that could affect the TMJ limits AI usability in specific real-life scenarios. Moreover, the applicability of many studies is potentially limited since the typical scenario in which an AI system might be used is for subjects presenting with the symptomatology of TMD, however, several studies did not specify how they assessed such groups. In addition, the use of a case-control design in one study could have induced a selection bias. Concerning the reference standard, although the majority of the studies used good reference tests such as CBCT (with one exception that used orthopantomography, which is known to have reduced accuracy), they usually did not specify the observer experience and how many different observers assessed the images, nor their intra- and inter-rater reliability, thus potentially reducing the confidence in the standard test. One study used human intervention in confirming the region of interest, which precludes the creation of complete functional pipelines but helps accuracy; however, the other studies used automated methods. In addition, the studies did not perform sensitivity analyses and only a few studies had an appropriate tier of transparency by sharing their code.

### 4.2. Study Strengths

Finding new non-invasive approaches to diagnose TMJ OA accurately, forecast illness severity, devise treatment plans, assess prognosis, and track disease progression is an important result that can be built upon this work. However, our study exposes significant gaps in the data that need to be investigated further in follow-up research while providing a neutral summary of the available literature. A key advantage of our study is the comprehensive search strategy combined with seven different databases. Furthermore, we used two instruments to assess the quality of the included papers. The first one, the QUADAS 2 tool, is endorsed by the Cochrane Collaboration, which is regarded as providing the highest level of evidence-based medicine worldwide. The second one, while not a quality assessment tool, is the only instrument that assesses the reporting information on clinical artificial intelligence modeling. Finally, since several studies used the same methods, we performed a meta-analysis to obtain their pooled results.

## 5. Conclusions

Our extensive literature search identified a rather diverse spectrum of AI applications on TMJ OA classification. Some studies focused on the diagnosis of TMJ OA using panoramic radiography with different transfer learning models, on which we performed a meta-analysis regarding the ResNet model. The other studies focused on CBCT images concerning its 3D shape or disease classification or combined the numerous radiomics features with clinical and proteomic data to explore the best models and promising features for TMJ OA diagnosis. The accuracies of the methods were similar overall and varied between moderate to good, being higher when excluding indeterminate diagnoses or when using fine-tuning. Future studies should employ better methods to amend the current literature papers’ limits.

## Figures and Tables

**Table 1:** Search strategies for each database.

PubMed
(“osteoarthritis” [MeSH Terms] OR osteoarthritis [All Fields] OR “Degenerative joint disease” OR (“degenerative” AND “joint” AND “disease”)) AND (“temporomandibular joint” [MeSH Terms] OR (“temporomandibular” [All Fields] AND “joint” [All Fields]) OR “temporomandibular joint” [All Fields] OR “TMJ” [Title/Abstract] OR “temporomandibular joint disorders” [MeSH Terms] OR (“temporomandibular” [All Fields] AND “joint” [All Fields] AND “disorders” [All Fields]) OR “temporomandibular joint disorders” [All Fields] OR (“temporomandibular” [All Fields] AND “disorders” [All Fields]) OR “temporomandibular disorders” [All Fields] OR “TMD” [Title/Abstract]) AND (“Artificial intelligence” [MeSH Terms] OR “Artificial intelligence” [All Fields] OR “machine intelligence” [All Fields] OR “Machine Learning” [MeSH Terms] OR “Machine Learning” [All Fields] OR “Deep Learning” [MeSH Terms] OR “Deep Learning” [All Fields] OR (“Learning” AND (“supervised” OR “unsupervised”)) OR “Support Vector Machines” [All Fields] OR “Random forest” [All Fields] OR “classifier” [All Fields] OR “classification algorithm” [All Fields] OR “cross validation” [All Fields] OR “data mining” [All Fields] OR “feature detection” [All Fields] OR “feature extraction” [All Fields] OR “feature learning” [All Fields] OR “feature selection” [All Fields] OR “k nearest neighbor” [All Fields] OR “pattern recognition” [All Fields] OR “KNN” [All Fields] OR “K-means” [All Fields] OR “Principal Component Analysis” OR “XGBoost” [All Fields] OR “LightGBM” [All Fields] OR “Neural Network” [All Fields] OR “Tensorflow” [All Fields] OR “PyTorch” [All Fields] OR “Keras” [All Fields] OR “ResNet” [All Fields])
EMBASE
(‘osteoarthritis’/exp OR osteoarthritis OR ‘degenerative joint disease’/exp OR ‘degenerative joint disease’ OR (‘degenerative AND (‘joint’/exp OR ‘joint’) AND (‘disease’/exp OR ‘disease’))) AND (‘temporomandibular’ AND (‘joint’/exp OR ‘joint’) OR ‘temporomandibular joint’/exp OR ‘temporomandibular joint’ OR ‘tmj’ OR (‘temporomandibular’ AND (‘joint’/exp OR ‘joint’) AND (‘disorders’/exp OR ‘disorders’)) OR ‘temporomandibular joint disorders’/exp OR ‘temporomandibular joint disorders’ OR (‘temporomandibular’ AND (‘disorders’/exp OR ‘disorders’)) OR ‘temporomandibular disorders’ OR ‘tmd’) AND (‘artificial intelligence’/exp OR ‘artificial intelligence’ OR ‘machine learning’/exp OR ‘machine learning’ OR ‘deep learning’/exp OR ‘deep learning’ OR ‘deep neural network’/exp OR ‘deep neural network’ OR ((‘learning’/exp OR ‘learning’) AND (‘supervised’ OR ‘unsupervised’)) OR ‘support vector machines’/exp OR ‘support vector machines’ OR ‘random forest’/exp OR ‘random forest’ OR ‘classifier’/exp OR ‘classifier’ OR ‘knn’ OR ‘k-means’ OR ‘principal component analysis’/exp OR ‘principal component analysis’ OR ‘xgboost’/exp OR ‘xgboost’ OR ‘lightgbm’ OR ‘neural network’/exp OR ‘neural network’ OR ‘tensorflow’/exp OR ‘tensorflow’ OR ‘pytorch’ OR ‘keras’ OR ‘resnet’/exp OR ‘resnet’)
Scopus
ALL ((“osteoarthritis” OR “degenerative joint disease” OR (“degenarative” AND “joint” AND “disease”)) AND ((“temporomandibular” AND “joint”) OR “temporomandibular joint” OR “tmj” OR (“temporomandibular” AND “joint” AND “disorders”) OR “temporomandibular joint disorders” OR (“temporomandibular” AND “disorders”) OR “temporomandibular disorders” OR “tmd”) AND (“artificial intelligence” OR “machine learning” OR “deep learning” OR “deep neural network” OR (“learning” AND (“supervised” OR “unsupervised”)) OR “support vector machines” OR “random forest” OR “classifier” OR “knn” OR “k-means” OR “principal component analysis” OR “xgboost” OR “lightgbm” OR “neural network” OR “tensorflow” OR “pytorch” OR “keras” OR “resnet”)) AND (LIMIT-TO (DOCTYPE, “ar”) OR LIMIT-TO (DOCTYPE, “re”)) AND (LIMIT-TO (SUBJAREA, “DENT”))
Web of Science
TS = ((“osteoarthritis” OR “degenerative joint disease” OR (“degenerative” AND “joint” AND “disease”)) AND ((“temporomandibular” AND “joint”) OR “temporomandibular joint” OR “tmj” OR (“temporomandibular” AND “joint” AND “disorders”) OR “temporomandibular joint disorders” OR (“temporomandibular” AND “disorders”) OR “temporomandibular disorders” OR “tmd”) AND (“artificial intelligence” OR “machine learning” OR “deep learning” OR “deep neural network” OR (“learning” AND (“supervised” OR “unsupervised”)) OR “support vector machines” OR “random forest” OR “classifier” OR “knn” OR “k-means” OR “principal component analysis” OR “xgboost” OR “lightgbm” OR “neural network” OR “tensorflow” OR “pytorch” OR “keras” OR “resnet”))
LILACS
tw:((“osteoarthritis” OR “degenerative joint disease” OR (“degenerative” AND “joint” AND “disease”)) AND ((“temporomandibular” AND “joint”) OR “temporomandibular joint” OR “tmj” OR (“temporomandibular” AND “joint” AND “disorders”) OR “temporomandibular joint disorders” OR (“temporomandibular” AND “disorders”) OR “temporomandibular disorders” OR “tmd”) AND (“artificial intelligence” OR “machine learning” OR “deep learning” OR “deep neural network” OR (“learning” AND (“supervised” OR “unsupervised”)) OR “support vector machines” OR “random forest” OR “classifier” OR “knn” OR “k-means” OR “principal component analysis” OR “xgboost” OR “lightgbm” OR “neural network” OR “tensorflow” OR “pytorch” OR “keras” OR “resnet”))
Proquest
(“osteoarthritis” OR “degenerative joint disease” OR (“degenerative” AND “joint” AND “disease”)) AND ((“temporomandibular” AND “joint”) OR “temporomandibular joint” OR “tmj” OR (“temporomandibular” AND “joint” AND “disorders”) OR “temporomandibular joint disorders” OR (“temporomandibular” AND “disorders”) OR “temporomandibular disorders” OR “tmd”) AND (“artificial intelligence” OR “machine learning” OR “deep learning” OR “deep neural network” OR (“learning” AND (“supervised” OR “unsupervised”)) OR “support vector machines” OR “random forest” OR “classifier” OR “knn” OR “k-means” OR “principal component analysis” OR “xgboost” OR “lightgbm” OR “neural network” OR “tensorflow” OR “pytorch” OR “keras” OR “resnet”); filters: article, peer-review, osteoarthritis
SpringerLink
“osteoarthritis” AND ((“temporomandibular” AND “joint”) OR (“temporomandibular” AND “disorders”) OR “TMJ” OR “TMD”) AND (“artificial intelligence” OR “machine learning” OR “deep learning” OR “neural network”); filters: article, Imaging/Radiology

## Data Availability

Data available on request from the corresponding author, upon reasonable request.

## Supplementary Materials

The following supporting information can be downloaded at: https://www.mdpi.com/article/10.3390/jcm12030942/s1, Supplementary Table S1: Study characteristics.
