# Supplementary material for: Temporomandibular Joint Osteoarthritis Diagnosis Employing Artificial Intelligence: Systematic Review and Meta-Analysis
Source: J Clin Med. 2023 Jan 25;12(3):942. doi: 10.3390/jcm12030942 (PMC9918072; doi:10.3390/jcm12030942)
Supplement: Supplementary file 1 [file jcm-12-00942-s001.zip › jcm-2165713-supplementary.pdf]

Supplementary Table S1. Study characteristics.

Author, year	Study population	OA classification	Training, validation, and testing	Extraction ROI	Transfer learning models	Learning	Software	Results
**Bianchi J, 2020 [34]**	Subjects enroled: January 2016 to December 2018, in two groups: TMJ OA and Control groups. Cross-sectional study. 92 patients, 46 TMJ OA and 46 age and sex-matched control subjects. Age 21–70 years, no history of cancer, jaw joint trauma, surgery, or recent jaw joint injections, systemic diseases; no current pregnancy and no congenital bone or cartilage disease. The Control group subjects were recruited by advertisement.	Radiographic criteria of Ahmad, M. et al.	10 times' 5-fold CV by taking 4 folds as training and the remaining one-fold as validation with 10 times' repetition	Manual ROI using the module "Crop-Volume" of the 3D-Slicer Software	Not used	The models were trained on 52 features (clinical features (age, years of pain, vertical range unassisted without pain, and others), 20 radiomics features (eg. energy, entropy, bone volume, trabecular thickness, and others), 14 serum and saliva biomarkers (Custom human quantibody protein microarrays from RayBiotech, Inc. Norcross, GA)). Logistic regression model, Extreme Gradient Boosting (XGBoost), Light Gradient Boosting Machine (LightGBM), and Random Forest models. AUC chosen as the evaluation criterion.	-	XGBoost +LightGBM model with these features and interactions achieves the accuracy of 0.823, AUC 0.870, and F1-score 0.823 to diagnose the TMJ OA status. hypothesize that by combining standardized patient features from multiple sources using statistical machine-learning approaches, we can accurately diagnose TMJ OA status
**Choi E, 2021 [38]**	Patients who visited the orofacial pain clinic of Seoul National University Dental Hospital who reported TMD-	1189 OPGs, all of which had	2378 joints were divided randomly into training (1478 images), validation	ROI including the mandibular condyle and	Karas' Inception	Data augmentation was done by image rotation ± 5 degrees,	Python programming	Results for the testing set including indeterminate TMJ-OA diagnosis:

	related symptoms and had an OPG and TMJ CBCT, from January, 2015 to October, 2019.<br>1189 Orthopantomograms images confirmed by CBCT were read by OMFR specialists.<br>Excluded: < 18 years of age or with a history of orthognathic surgery, macro trauma, and systemic diseases.	been confirmed by additional CBCT examination, selected randomly and classified by an orofacial pain specialist in terms of image analysis criteria for the diagnosis of temporomandibular disorder (research diagnostic criteria for TMD14; diagnostic criteria for TMD15: no TMJOA (normal), indetermin	(450 images), and test sets (450 images). The test set consisted of 150 normal, 150 indeterminate, and 150 OA images	surrounding structures from each OPG identified by object detection. Faster RCNN using the Inception V3 model as the categorizing algorithm in which Region Proposal Network and Image Classification Network work simultaneously and make it faster. The RCNN used extracted ~2000 region proposals. For each region, 4096-dimensional feature vectors were derived using CNN for image classification (Inception ResNet V2r) were warped to a size of 227 × 227 color image that the CNN used as the input and derived the included	ResNet V2	image shift ± 10%, brightness ± 10%, and contrast ± 10%. Training and validation were repeated 35,000 times (700 epochs) with augmented data. An Adam optimizer was used. After 700 training epochs, the validation loss of the model decreased from 12.2 to 0.1. Five-fold cross validation was performed to evaluate model training using an 80 to 20 percentage split.	language (v. 3.6), Tensorflow (v. 2.0) and a graphics card (GeForce GTX 2080) were used for analysis	accuracy=0.51, weighted average precision=0.55, weighted average recall=0.51, F1 score=0.53. Results excluding indeterminate TMJ-OA diagnosis: accuracy=0.78, weighted average precision=0.78, weighted average recall=0.78, F1 score=0.78.

		ate for TMJOA (indeterminate), and TMJOA (OA).		characteristics through 5 convolutional layers and 2 fully-connected layers. Then, a SVM was used to predict the class. Finally, Bounding-box regression was performed to determine the location of the objects more accurately				
**de Dumast P, 2018 [35]**	Subjects recruited from the university clinic and through advertisement. For the image analysis classification, 34 right and left condyles from 17 patients (39.9 ± 11.7 years), who experienced signs and symptoms of the disease for less than 5 years, were included as the testing dataset.	Radiographic criteria of Ahmad, M. et al.	The training dataset consisted of 259 condyles, 105 from control subjects and 154 from patients with diagnosis of TMJ OA	Constructed 3D surface models from the CBCTs using segmentation with ITK-SNAP 2.4 software. Left condyles were mirrored in the sagittal plane to be in the same orientation as the right condyles to facilitate bilateral comparisons. All condylar models were simultaneously	Not used	The SVA module of the Slicer software was used for automatic classification of morphological variation in TMJ OA, based on a neural network with an architecture of 1 hidden layer, 2001 iterations, 50 epochs.	ITK-SNAP 2.4, SPHARM-PDM, 3D Slicer software, TensorFlow	Accuracy of TMJ-OA degeneration from confusion matrix categories 14/23 = 0.609. 91% agreement between the clinician and the SVA classifier

				cropped to obtain ROI. SPHARM-PDM software was used to generate a mesh with 1002 correspondent vertices.<br>An average 3D condylar shape was generated using the ShapeVariationAnalyzer (SVA) extension for the 3D Slicer open-source software.				
**Jung W, 2021 [39]**	Patients that visited the Department of Oral Medicine, Jeonbuk National University Dental Hospital from January 2015 to December 2018 for pain in the TMJ area.<br>518 subjects TMJ-OA. 1118 patients with panoramic TMJ radiography and TMJ CBCT.<br>Excluded: subjects with other bone lesions, fracture of the condyle, as well as illegible or blurry images.	Radiographic criteria of Ahmad, M. et al. and Schiffman et al.	The 858 images included, 395 normal images and 463 TMJ-OA images. The data were randomly divided into training, validation, and evaluation sets (6:2:2).	A data preprocessing platform was used to select the ROI. The image was uploaded to the platform and manually cropped. Manual selection of the ROI, which included the condyle by several specialists.	Resnet152 and EfficientNet-B7	Training was conducted under various conditions. The training parameters of the best model with the smallest validation loss in the learning process included a learning rate of 10−4, 1000 epochs, an Adam optimizer, and a weight decay of 10−4.	Anaconda4.9.2, Python3.8, Pytorch1.8.1, cuda11.1, and GPU RTX 3090	ResNet-152 accuracy = 0.87. The Se, Sp, AUC values were 0.94, 0.79, and 0.94. EfficientNet-B7 accuracy = 0.88. The Se, Sp, AUC values were 0.86, 0.91, and 0.95.
**Kim D, 2020 [40]**	The patient group received treatment in one of the hospitals affiliated with the AIQUB Dental Network in South Korea between July and September 2018.	no information	Several algorithms were developed: to detect the TMJ and joint fossa and condyle (Model 1: R-CNN model, 800 labeled images	Model 1 and 2 were used for the condylar ROI detection	VGG16, ResNet, and Inception V3	Model 3 was set to determine the presence or absence of TMJ OA 923 and 231 images as training and test sets.	PyTorch	VGG16 accuracy=0.78, Se=0.49, Sp=0.86, AUC=0.76. ResNet accuracy=0.77, Se=0.41, Sp=0.77, AUC=0.57.

	1292 patients aged between 20 and 60 (700 men, 592 women, average age: 43.3 years).		for training and 187 images as test data); to detect abnormalities based on the shape of the TMJ (Model 2: applied the CNN algorithm to the condylar region extracted using the results from the first model and determined whether the extracted condyle was appropriately photographed, using 2066 and 518 images as training and test sets)					Inception V3 accuracy=0.79, Se=0.39, Sp=0.82, AUC=0.51.<br>Fine tuning: VGG16 accuracy=0.84, Se=0.54, Sp=0.94, AUC=0.82. ResNet accuracy=0.81, Se=0.47, Sp=0.91, AUC=0.79.<br>Inception V3 accuracy=0.82, Se=0.41, Sp=0.94, AUC=0.83.
**Lee KS, 2020 [36]**	314 patients with TMD and signs of TMJOA on CBCT were included (84 males, 230 females; mean ± SD age, 39.5 ± 18.2 y; range, 16 to 84 y). From the prepared 3,749 images, 235 identified as normal were excluded. 1,700 were diagnosed as indeterminate for TMJOA, and 1,814 TMJOA.	Radiographic criteria of Ahmad, M. et al. and Schiffman et al.	Trained with the 3,514 images and tested with 2 sets of 300 images - each including: 150 images: 50 cases of normal TMJ and 50 cases control group, and 50 cases of TMJOA group. Half of the 3,514 images were used for building the model, and the other half for validating the model.	Manual extraction	Not used	Single-shot detector (SSD) deep learning framework designed for object detection, (with 10,700 epochs). The learning rate of the model was 1.0 × 10–5 during the first 450 epochs and 1.0 × 10–4 during the last 10,250.	Python	Results for the testing set including indeterminate TMJ-OA diagnosis: set average precision=0.80, set average recall=0.77, F1 score=0.78. Results excluding indeterminate TMJ-OA diagnosis: set average precision=0.89, set average recall=0.90, F1 score=0.89.
**Zhang W, 2021 [37]**	Same group as for Bianchi J, 2020 study<br>Subjects enroled: January 2016 to December 2018, in two groups: TMJ OA and Control groups.<br>Cross-sectional study.<br>92 patients, 46 TMJ OA and 46 age and sex-matched control subjects.<br>Age 21–70 years, no history of cancer,	Radiographic criteria of Ahmad, M. et al.	80%–20% training testing split, and 5-fold cross-validation on the training set	Each HR-CBCT volume was cropped manually into a ROI greyscale sample of 50 × 50×50 slices (voxel size of 0.08 mm3) containing the	3D-Slicer	Dataset consists of 77 features (6 clinical, 46 imaging, and 25 protein) collected from 92 patients. 3 non-LUPI algorithms (Gaussian SVM, RVFL, and Adaboost), compared to the	-	In all comparisons, LUPI outperforms non-LUPI methods. Accuracy increased by at least 2.2% for all 3 comparisons. AUC and Accuracy SD decreased for IPL and KRVFL+ models. All comparisons had an

jaw joint trauma, surgery, or recent jaw joint injections, systemic diseases; no current pregnancy and no congenital bone or cartilage disease. The Control group subjects were recruited by advertisement.		trabecular/cortical condyle bone without extrapolated the condyle boundaries, using the module “Crop-Volume” of the 3D-Slicer Software.	performance of 3 LUPI-based algorithms (SVM+, KRVFL+, and IPL)	increase in performance after including protein interaction features. Comparing PrX LUPI with non-LUPI performance from the previous experiment, SVM+ improved AUC by 0.027 and accuracy by 2.7% when compared to SVM, IPL improved AUC by 0.018 and accuracy by 7.3% when compared to Adaboost, and KRVFL+ improved AUC by 0.056 and accuracy by 5.6%.

TMJ OA, temporomandibular joint osteorarthritis; CV, cross-validation; ROI, region of interest; AUC, area under the curve; TMD, temporomandibular disorder; TMJ, temporomandibular joint; CBCT, cone bean computed tomography; OMFR, oromaxilofacial radiologist; OPT, orthopantomogram; RCNN, regions based convolutional neural networks; SVM, support vector machine; SVA, shape variation analyser; VGG, Visual Geometry Group; ResNet, Residual Networks; Se, sensitivity; Sp, specificity; F1 score = 2*precision*recall/(precision+recall); LUPI, Learning using Privileged Information; IPL, iterated privileged learning model; RVFL, random vector functional link network; KRVFL, kernel-based RVFL; PrX, protein interaction set.
